# 678. Rapid Molecular SARS-CoV-2 Detection by Abbott ID NOW Is Reliable in Pediatric Patients

**DOI:** 10.1093/ofid/ofab466.875

**Published:** 2021-12-04

**Authors:** Catherine Murphy, Emily Sheboy Scarcello, Sheila M Nolan

**Affiliations:** 1 NYMC, Valhalla, New York; 2 BCHP, Valhalla, New York; 3 New York Medical College, Valhalla, New York

## Abstract

**Background:**

The COVID-19 Pandemic demonstrated the importance of rapid, accurate, point of care testing to control spread of the virus. The availability of this testing has been crucial to re-opening schools, keeping children safely in schools, and returning children to school quickly following illness. The Abbott ID Now molecular assay to detect SARS-CoV-2 was granted Emergency Use Authorization in March 2020. Reports of lower sensitivity compared with conventional PCR prompted some school districts to require confirmatory conventional PCR for negative rapid molecular results to return children to school. In this study we aim to determine the sensitivity and specificity of the Abbott ID NOW molecular SARS-CoV-2 test in a large pediatric primary care practice.

**Methods:**

A retrospective observational study was performed using data from 25 pediatric primary care sites in the Boston Children’s Health Physicians network, a large multispecialty pediatric practice in New York and Connecticut. Data were extracted from the electronic health record for all patients 0-22 years of age who had an Abbott ID NOW rapid molecular COVID-19 assay from October 1, 2020 - February 28, 2021. For all patients with rapid tests, we identified patients who had a conventional PCR test sent within 1 day before or 1 day after the ID NOW test. The result of the conventional PCR test was considered the “true” result. All discrepant test results were identified.

**Results:**

During the study period, 14993 patients had ID NOW testing performed. The percent positivity was 8.5%. The percent positivity in our practices paralleled that in the surrounding community throughout the winter surge of COVID-19. 500 patients had confirmatory testing sent within 1 day before or after the ID NOW test (15 positive and 485 negative results). Based on the conventional PCR test results, 2 of 15 positive results were false positive and only 1 of 485 negative results was a false negative, resulting in a sensitivity of 93% and specificity of 99.6%. The false negative result was in a patient with nasal congestion whose mother was COVID positive.

**Conclusion:**

Rapid, molecular, point of care testing is an important tool to identify SARS-CoV-2 in pediatric patients and limit school absences. The ID NOW assay is highly sensitive and specific in a real-world pediatric setting.

**Disclosures:**

**All Authors**: No reported disclosures

